# Autophagy takes the STING out of DNA sensing

**DOI:** 10.1038/s41423-021-00797-3

**Published:** 2021-11-02

**Authors:** Carlos Maluquer de Motes

**Affiliations:** Department of Microbial Sciences, University of Surrey, Guildford, GU2 7XH UK

**Keywords:** Autophagy, Innate immunity

**Fig. 1:**
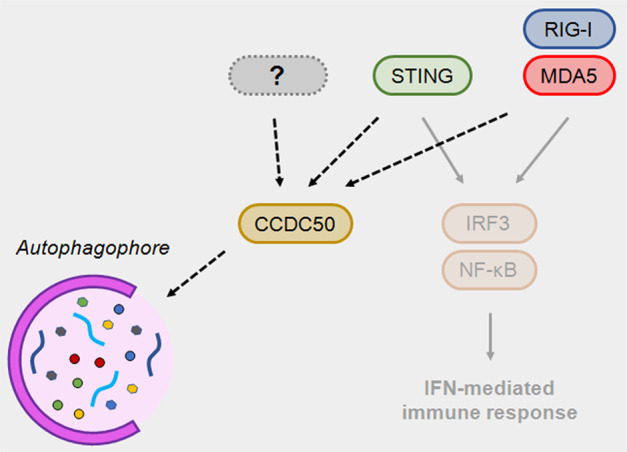
The role of CCDC50 in suppressing the IFN-mediated immune response. Foreign nucleic acids trigger a potent immune response characterized by the production of inflammatory mediators, including type I interferon (IFN). Foreign RNA is recognized by RNA immune sensors such as retinoic acid inducible gene I (RIG-I) and melanoma differentiation-associated protein 5 (MDA5). Cytosolic DNA can be recognized by cyclic GMP-AMP (cGAMP) synthase (cGAS), which in turn activates stimulator of IFN genes (STING). Signal transduction from these sensors converges to activate the immune transcription factors IFN responsive factor 3 (IRF3) and nuclear factor κ-light-chain-enhancer of activated B cells (NF-κB). These transcription factors drive the expression of hundreds of genes, including cytokines and chemokines and their modulators, immunoreceptors and stress response genes. Coiled-coil domain-containing protein 50 (CCDC50) is an autophagic cargo receptor that directs polyubiquitin-activated STING, RIG-I, and MDA5 toward nucleated phagophores and drives their autophagic degradation. This negative regulation results in reductions in IFN and innate responses

The innate immune system has evolved to detect molecular signatures associated with invading microbes and the cellular damage these microbes cause. Recognition of microbial genomes, particularly those of intracellular microbes such as viruses, is crucial in triggering a protective response and preventing infection. Many nucleic acid forms are uniquely associated with pathogens (e.g., 5’ppp-RNA, dsRNA) or localize in abnormal cellular compartments during infection (e.g., cytosolic DNA), facilitating the distinction between self and nonself [1]. In particular, the detection of cytosolic DNA has emerged as an important process in infection, as well as cancer, sterile inflammation, and autoimmune diseases such as systemic lupus erythematosus (SLE), in which self-DNA induces type I interferon (IFN) signaling and increases the expression of IFN-stimulated genes (ISGs). Although it has been known for decades that foreign DNA activates potent immune responses [2], only recently have we started to identify the proteins that are responsible for these responses and how they work [3]. A crucial molecule in this process is stimulator of interferon genes (STING), an ER/Golgi-resident protein that recognizes cyclic GMP-AMP (cGAMP), the product of the activated DNA-binding enzyme cGAMP synthase (cGAS). Activation of STING results in a potent transcriptional response involving IFN and multiple inflammatory genes. To ensure that immune responses are proportionate and can eliminate the exogenous challenge without inducing excessive immunopathology, a fine balance between activation and deactivation must be maintained [4]. Writing in *Cellular & Molecular Immunology*, Hou et al. [5]. revealed how activated STING is delivered for degradation by the autophagic cargo receptor CCDC50, illuminating the mechanisms that control STING turnover and regulate immune responses to infection and chronic autoimmune disease (Fig. 1).

Autophagy is an essential process that mediates the breakdown and recycling of unwanted cargo and cellular organelles. Autophagy is an integral part of many cellular stress responses, including those induced during infection and inflammation. Autophagy modulates both the induction and suppression of immune and inflammatory responses, and this complex relationship is likely to be determined by mechanisms associated with the selective recognition of substrates. Cellular cargo is typically directed to autophagosomes by autophagy adaptors such as p62, NBR1, or NDP52. These adaptors recognize polyubiquitylated substrates via ubiquitin-binding domains (UBDs) and target these substrates to nascent phagophores via association with the membrane protein LC3 (ATG8). CCDC50 (also known as Ymer) shares similarities with these adaptors. CCDC50 contains a UBD that recognizes polyubiquitylated substrates and LC3-interacting regions (LIRs). Notably, both canonical and the recently identified, noncanonical [6] LIRs are present in CCDC50, allowing it to dock on 2 different regions on LC3. What regulates the use of one LIR or another remains to be determined. The authors show that the motif interacting with ubiquitin (MIU) domains in CCDC50 recognize K63-polyubiquitylated STING. CCDC50 was previously shown by the same group to downregulate IFN-inducing RNA sensors such as RIG-I and MDA-5 [7]. This finding suggests that CCDC50 targets other immune sensors and has a larger repertoire of substrates that is possibly determined by the stress response in the cell. A proteomics inventory will highlight the molecular determinants that mediate CCDC50 substrate recognition. Interestingly, while CCDC50 promotes the association of the RNA sensors RIG-I and MDA-5 with the well-studied autophagy adaptor p62 [7], the role of CCDC50 in STING downregulation appears to be independent of p62, despite p62 having been previously associated with STING downregulation [8]. This poses the question of whether seemingly redundant adaptors may act cooperatively under certain stress conditions or in certain tissues and/or once an activation threshold is surpassed.

In agreement with its role in immune regulation, the authors showed that CCDC50 was highly expressed in immune cells, including plasmacytoid dendritic cells (pDCs). IFNs are produced at very low levels in nonpathological conditions but are rapidly upregulated upon activation of several innate immune signaling pathways, particularly in pDCs, which are equipped with multiple immune sensors. SLE is an autoimmune disease that is characterized by autoantibody production and severe inflammation that results in fatal glomerulonephritis. SLE pathogenesis is notoriously complex and heterogeneous, but IFN and IFN-producing cells such as pDCs are known to play significant roles in this disease, and many patients exhibit enhanced ISG expression signatures [9]. By analyzing publicly available gene expression databases and peripheral blood mononuclear cells from SLE patients, Hou et al. demonstrated that CCDC50 expression is downregulated and negatively correlates with IFN levels and SLE pathogenesis markers. These results identify CCDC50 as a important new player in SLE and related diseases and question the regulation of CCDC50 expression and activity under biological and pathological conditions. In addition, the estimated incidence, prevalence and mortality of SLE vary considerably between geographic regions; age and sex (SLE predominantly affects young women); and ethnicity, with certain ethnic groups experiencing increased morbidity and mortality. The molecular mechanisms that govern SLE pathogenesis, including the positive and negative regulation of intracellular signaling pathways, may differ in these populations and need to be examined. Although progress in the development of SLE therapies has been slow compared to similar pathologies, new exciting approaches offering hope for the approval of new therapies are on their way and include the targeting of cytokines and signal transduction pathways.

IFN responses are critical for the initiation of anticancer immune responses, and accordingly, significant effort has been made by the pharmaceutical industry to produce effectors that stimulate STING signaling for use in oncological therapeutic regimes. Although more than 15 different STING agonists have been developed, no phase III trials have yet been launched due to disappointing clinical benefits. These outcomes point towards tumor-specific immune evasion strategies that neutralize STING. Epigenetic silencing of cGAS and STING has been reported in various cancer types [10]. The findings from Hou et al. suggest that posttranslational autophagic turnover of STING may also be at play. The expression of CCDC50 and regulation of its activity in cancer tissues deserve further investigation and may provide novel insights to strategically boost antitumor immunity. Collectively, this work expands our current understanding of STING regulation and may have far-reaching implications for the therapeutic manipulation of STING signaling.
